# Monograph of the First Permanent Molar

**Published:** 1888-03

**Authors:** E. Audrieu

**Affiliations:** Paris


### Monograph of the First Permanent Molar.

BY DR. E. AUDRIEU, PARIS.

(Translated by G. P. Terry.)

A SYNOPSIS.

*Continued from page 534, Vol. XLI.*

### CAUSES OF THE FREQUENCY OF THE DECAY OF THE WISDOM TOOTH.

We have mentioned before, that after the first permanent molar the wisdom tooth decays the most frequently. This proposition is admitted by almost all dentists; but yet it is not always so, and it is, in fact, absolutely dependent in the most of cases on the presence of the first permanent molars, and especially in mouths with arches too small to contain all the teeth. We believe this to be the explanation of this frequency of decay.

At the period of the eruption of the second permanent molars all the permanent teeth, less the second bicuspids, are in place.

The second permanent molar has, therefore, more space to resist decay than the first permanent molar, hence the causes of acidity of the saliva which we have pointed out, do not, so to say, exist; but it is not the same with the wisdom tooth. In the lower jaw, for example, when, in consequence of the presence of all the other permanent teeth at the time of eruption of the third molar, the jaw is too small to contain all the teeth, and that the wisdom tooth is crowded between second permanent molar and anterior part of the ramus of the maxillary bone, is not able to make its eruption in a normal state, there happens a phenomenon very important in regard to the health of that tooth. Its crown, which cannot completely emerge, shows only its two anterior cusps, and the eight-tenths of its grinding surface remain covered by a small tongue of tissue, which acts as a cover in this way during six months, a year, and even more.

But, during this time, there exists between the tooth and its covering a receptacle more or less considerable for food-debris which no washing can remove, from which ensues acid fermentation, destruction of enamel and decay.

In the upper jaw the condition is the same, but the mechanism is different.

The wisdom tooth has always room to accomplish its eruption, since nothing hinders back of it; but in the conditions of too small a dental arch, instead of growing down vertically, it takes an oblique course, its masticating surface more or less turned backwards, from which results a faulty position, where foods are intruding easily between the wisdom tooth and the second permanent molar, and in this inter-dental space lodge, acidify, and have a deleterious effect upon the contiguous teeth, the action being more powerful upon the wisdom tooth than on its neighbor, for the following reason:

The wisdom tooth in these cases has but relatively weak, short, conical roots, it gives way to the pressure of foods, and gets out of its position and leaves a space always filled with food, which is almost impossible to cleanse. It is there indeed that decay is found. From which we can draw this conclusion, that the wisdom tooth, when its eruption is hindered by a want of room, be it of the relative shortness of the dental arch, be it of the too great volume of the other teeth in relation to this length, is the most often decayed, a conclusion which is corroborated by this second proposition, which is now to be proven.

If the first permanent molar had been extracted, (and we will see later on why we mean the first permanent molar, and not another,) and that in consequence of this extraction the wisdom tooth has had sufficient room to erupt, then it, finding itself through, decays very rarely.

The two following statistics, made about ten years ago, suffice certainly to prove this well-founded.

CITY PRACTICE.

One hundred mouths (irrespective of sex), from twenty-five to thirty years old.

| First per. molars present in health, filled, decayed. | LEFT SIDE. | | RIGHT SIDE. | |
|---|---|---|---|---|
| | Wisdom Teeth Decayed. | Wisdom Teeth Healthy. | Wisdom Teeth Decayed. | Wisdom Teeth Healthy. |
| Lower jaw | 73 | 27 | 71 | 29 |
| Upper jaw | 62 | 38 | 57 | 41 |

CITY PRACTICE.

One hundred mouths, from twenty-five to thirty years old.

| First per. molars absent. | LEFT SIDE. | | RIGHT SIDE. | |
|---|---|---|---|---|
| | Wisdom Teeth Decayed. | Wisdom Teeth Healthy. | Wisdom Teeth Decayed. | Wisdom Teeth Healthy. |
| Lower jaw | 16 | 84 | 13 | 87 |
| Upper jaw | 10 | 90 | 11 | 89 |

The wisdom tooth is the replacement tooth of the permanent first molar.

Dwelling on these figures, and we can add, upon the less precise observations, it is true, we believe, we are able to express the opinion, that the wisdom tooth, although it does not come to occupy the space left by the first permanent molar before its extraction, and although it be separated from that space by the second permanent molar, is, nevertheless, in a practical point of view, the replacement tooth of the first permanent molar, which is destined to decay.

CONDITIONS OF EXTRACTION OF THE FIRST PERMANENT MOLAR.

There remains for us to indicate the practical consequences which one can draw from the considerations which we have developed, that is the, conditions of extraction of the first permanent molar.

We will pass rapidly the principal points in view.

But let us begin by solving a question whose solution is evidently *a priori.*

When the milk teeth are of good quality, when they are shed in their time, without being decayed, when the first permanent molars are well formed and their grinding surface healthy, when the jaws appear to be of sufficient dimensions to contain all the permanent teeth, when at last the dental arches have a normal conformation, that is, when the superior is a full, round arch, and not pointed, and when the inferior is of proper diameter, it is very evident, and we insist upon this point, that unless, under exceptional circumstances, there can be no question in such a mouth of the extraction of the first permanent molar.

But one must not be mistaken. These exceptional cases are very rare, and by far more frequent are those where the deciduous teeth, extremely close together, leave but an insufficient space for the permanent teeth to arrange themselves where the milk molars decay rapidly and fall in "detritus," after having occasioned abscess after abscess, and where the first molar decays some months, a year, or two years after its eruption, and requires the helps of art.

These latter cases are the only ones too that must occupy us here, and we will point out the principal circumstances where it is truly rational to extract the first permanent molar.

It is first the decay of that tooth, then the regulation of teeth, in certain mouths of various conformations and of abnormal dental eruption.

### DENTAL DECAY.

As a rule, one can say that when a first permanent molar is affected by soft decay, whitish, chalk-like, when the other teeth are healthy, and before the second molar has achieved its eruption, it is preferable to extract it rather than to try to preserve it.

In fact, by extracting it in time, the second permanent molar encroaches little by little, without inclining forwards upon half the space which it occupied, and the second bicuspid the other half. Soon the space is all occupied, the other teeth spread themselves, their position is regulated, and the wisdom tooth will accomplish its eruption correctly.

If at an early age, instead of extracting the first molar it is filled, it is rare that the filling, with whatever ability and care it had been put in, would remain long. Soon the enamel which surrounds the filling is disengaged, the decay continues and the filling falls out wholly. One must not, when the pulp has been exposed, dream of exterpating it, and of washing the pulp canal to the bottom, it is very difficult indeed to determine while practicing the operation upon that tooth whether a periostitis and abscess will occur.

It may be regarded as a rule, although there are some exceptions, that a first permanent molar which decays between eight and eleven years, whatever care is taken to preserve it, decays on, little by little, either around the filling, or in other places, till it is destroyed. If the decay was in the molar alone it would not be so serious; but it is not so. If it decays upon its anterior contiguous surface, as the decay, notwithstanding the most scrupulous care continues its ravages, it is almost certain that the second bicuspid will suffer by means of contact. Does it decay upon its posterior contiguous surface it is the second molar which will be affected in its turn.

One would have then in this way, instead of one, three decayed teeth, which could have been avoided by one sure stroke, say the sacrifice of the first decayed tooth, in time, that is, the first permanent molar.

### THE REGULATING OF TEETH IN CERTAIN MOUTHS.

In regard to the regulating of teeth in certain mouths of abnormal or deformed dental eruption there is a rule from which we never depart because it is the fruit of a long experience; it is as follows:

All regulating which demands not more than from one to three months at the maximum, not only for obtaining the result but also for maintaining, without the extraction of teeth to effect the purpose, can and must be done with regulating apparatus alone.

That period is rarely sufficient to cause any injury to the teeth which serve as a prop or support to the apparatus. All regulating, on the contrary, which can not be obtained or maintained in less than three months, by the action of the apparatus without recurring to extraction, if the sacrifice of one or two teeth can regulate the arch in the preceding case, must be effected by extractions. Appliances which have to be worn in the mouth more than three months are pernicious to the teeth serving as props, partly on account of the tension or pressure exercised upon these teeth and partly on account of the uncleanliness of certain children, who at that age do not take any care whatever of their mouths.

This implies that when in regulating, one or more extractions are necessary, we make it a point to prove that it is the first permanent molar which should be extracted.

That the first permanent molars are the ones to be removed, which we make it a point to prove.

There are a great many practitioners who assert, that in leaving nature to act, or by helping her only by extracting milk teeth, whose shedding do not take place as soon as the permanent teeth appear, and leaving these latter to get into position themselves, that there are eight chances out of ten, that at a given moment regulation goes in to effect, thanks to the efforts of nature.

But we do not partake of that opinion, and moreover, are convinced that if, as we have experienced for more than twenty years at the "hospies des Enfants Assistes" they had noticed the eruption of teeth, in a considerable number of healthy mouths, they would change their opinion.

According to our observations, in almost half of the cases, one or more of the permanent teeth erupted out of position, or rather we found in each of these cases, one or more teeth placed on the outside of the row, without being able to get into position of themselves, as one of the anterior teeth of the upper jaw erupting so as to articulate in back of the teeth of the lower jaw, and also in consequence of the shortening of the arch, an incisor or canine of the lower jaw is being pressed forward in front of the upper teeth.

In these cases and in many others we think that it is better to let nature act. Neither should one, as Miel, a well-known dentist of Paris, act always in the same direction, clearing away the space and prematurely sacrifice almost all the milk teeth to allow the replacement teeth to erupt at their ease.

This method is not better than the preceding one, although there is yet the advantage of maintaining children's mouths clean and in consequence of this, of avoiding within certain limits the decay of the permanent teeth one must by a clear appreciation of circumstances, know how to take the middle course, follow attentively the phenomema of replacement and act at the right moment.

Let us suppose, and here is a frequent case—a narrow mouth —crooked, narrow, pointed arch, with the milk teeth of restrained volume in relation to those of replacement, and very near together, how must one act at the period of replacement?

In the lower jaw, if the two permanent central incisors fall back of the corresponding temporary ones the latter must be extracted. A little later the two permanent lateral incisors break forth in back of the two corresponding later temporary teeth but instead of erupting in position they are turned on their axis so as to show their ends more or less, that is, more or less in profile. What must be done? Immediately sacrifice the temporary incisors and also, although prematurely, the two deciduous canines. It is the only way of getting natural regulation of the two permanent lateral incisors, without artificial appliances.

One must act according to the same rule if one or two central incisors instead of presenting full face, as we have supposed it should present its profile, that is to say sacrifice immediately the two lateral temporary incisors. If the permanent incisors show only a little of their face it is useless, and even injurious to extract the temporary neighbors, unless the jaw be of exceptional narrowness. What will be the effect of these premature extractions?

The first bicuspid, which erupts always before the permanent canine, will have a tendency to encroach upon the space left unoccupied by the extraction of the temporary canine which, when the permanent canine appear, will cause necessarily a jog. If, in this case, as the permanent canine erupts before the second bicuspid, one extracts prematurely the second milk molar, it will probably happen that the first bicuspid instead of encroaching upon the space left free by the temporary canine, will direct itself backwards towards the first permanent molar, from which we draw forth the following rule: If having been obliged in order to regulate the permanent lateral incisor, to extract the neighboring temporary canine, we must, in order to hinder the first bicuspid from taking the place of the canine, sacrifice prematurely the second temporary molar and also furnish to the first bicuspid a free space toward which the pushing of the permanent canine will oblige it to direct itself.

These are the surest, easiest, shortest and safest means in regard to the other teeth, of regulating the eight anterior teeth, which by the laws of the most elementary dental esthetics, is of the highest importance.

We will soon see that concerning the back teeth their good order is not more difficult to obtain.

At this period the jaw is supplied, or at least the one we have operated on, with,

1. The second incisors.
2. The first bicuspid, grown almost up to its length, and moving towards the space left free by the extraction of the second temporary molar.
3. The canine showing only yet its cusp.
4. The first permanent molar.

We will soon come to the critical moment of the regulating. The second permanent molar erupts a little after the second bicuspid. One being back and one in front of the first permanent molar.

But the space destined for the second bicuspid has been encroached upon about three-quarters by the first bicuspid, and the second bicuspid directs itself inwards, and rarely outwards. On the other part the first permanent molar is not of good quality, is decayed. Is it not rational in such a case to extract that tooth. The space which it will free will soon be taken up, half by the second bicuspid and half by the second permanent molar, and if there remains for some time yet a small space between the teeth, it will be taken up later, thanks to the pressure exercised upon the second molar by the evolution of the wisdom tooth.

We have supposed that the first permanent molar was decayed, because this is the case most frequently; but it can also be healthy, and in such cases, what would be best to do?

There are two solutions to this problem.

1. The extraction of the first bicuspid. That is the classical solution.

2. The extraction of the first permanent molar. In this case the first solution affords a more rapid result. We only insist upon this rule when the regulating of a denture demands the sacrifice of a tooth, which is always the first permanent molar when it is decayed or perforated, which is the only thing convenient to do.

All this relates to the lower jaw; what must we now pursue for the upper jaw?

The large permanent incisors can erupt either in front or in back of the row of temporary teeth. When they erupt in front it suffices to extract the corresponding temporary teeth, and they will take proper place themselves. If they erupt back of the temporary teeth and threaten to descend back of the incisors of the lower jaw, the two temporary lateral incisors must be prematurely sacrificed, and immediately the two large incisors will gain their normal position without any aid.

But soon the permanent lateral incisors are going to erupt; if they appear in proper position and do not lift the lip too much nothing is to be done, we must wait; but if they are too far out in front, if they are turned or rather if they erupt so far back that a deformity for the central incisors is feared, the two temporary canines must immediately be sacrificed, and they will fall back into the case with the lower jaw, that is, that the first milk molar being about to shed, the second milk molar must immediately be sacrificed, so that the first bicuspid directs itself backwards than forwards, then later, as a definite solution for the order of the denture of the permanent first molar.

This is a method, which, if judiciously followed, has given us relatively easy success, while the other methods, which we have almost all tried, have taken much more time, and given difficulties, without more satisfactory results.

This example, which we have chosen as a typical one, with the end in view of proving the opportunity of extraction of the first permanent molar, for regulating the denture, in certain forms of mouths, suffices largely for alleging it; but there are numbers of other cases where this treatment is indicated just as well.

Thus, in the protrusion of the lower jaw the doing away with the first permanent molars of that jaw not only aids in regulating the denture but also, which is no less important, maintains it during the period of the eruption of the permanent third molar. Also in cases where the considerable volume of the teeth of one jaw stands in relation with that of the other, also in many others, yet too long to enumerate, and which the experienced dentist can easily recognize, and saying all in one word, in all those cases where the extraction of one or more teeth is indicated as favorable to the good order of the rest of the teeth.

What is the precise moment when this operation must be made, to get all the benefits possible out of it? This is easy to decide from the considerations stated before upon the physiological role of the first permanent molar. It is the moment when the second permanent molars not having erupted, or having but imperfectly done that, when the first bicuspids have attained their full length and are able to fulfill two of the roles of the first permanent molar, namely: The maintaining of the height of the articulation, and the accomplishment of mastication.

Earlier it will be injurious to these two functions. Later it leaves a space which the eruption of the wisdom tooth cannot close up fully. That is to say implicitly, it tends to preserve as much as possible the first permanent molar until the period of the election of its extraction. When it is attacked by decay it must be cared for from the very beginning, filled temporarily in a manner to let it fulfill as well as possible its role as a tooth of transition.

Such are the facts and deductions which have seemed to us worthy of notice in support of our mode of practice on the subject of the extraction of the first permanent molars.

At the beginning of our career two facts struck us continually then, by the examination of the mouths of our clients, facts which Dr. Delabarre had also remarked, but without drawing important conclusions from it.

We observe that in persons from thirty to forty years old who had lost their first permanent molars which had, according to their statement, been pulled out in youth, that the other teeth were always of healthy quality, whereas our young patients from fifteen to twenty years of age who had preserved their first permanent molars, that these teeth being good or decayed, had in general the rest of their teeth in bad condition. An idea struck us that there was some relation between these two facts, and we made researches in this direction.

The reason was simply this: twenty years or so before our entering into the profession, the only treatment applied to painful, decayed teeth, with very few exceptions, was extraction, and that the first permanent molar not being any better then it is now, was generally the one to be removed.

There was indeed no more decay proximate in the second permanent molar, or the second bicuspid; there was no generalized decay resulting from the acidification of the saliva caused by the decay of the first permanent molar, a kind of vicious center from which the health of the denture hardly escapes unimpaired. In one word, the other teeth being more free, arranged themselves conveniently. The wisdom tooth was healthy, and the health of the mouth was markedly better, whereas, when we began to practice the art of dentistry, the fashion, which in dentistry as well as in other things, claimed that no more extractions should be made.

It was, they said, a barbaric operation, and the dentist who practiced extraction was nothing more than a tooth-puller.

They had at all cost to be filled to preserve them whole, even if they had to be wholly built up.

It was then, that we taking fully in account that the right way was between the two extremes, we returned to the extraction of the first permanent molar, not to extraction universally, but to extraction guided and based upon the rational principles which we have set forth in this memento.

---

Type in School Books.—The Austrian Minister of Public Instruction has issued a decree forbidding the use in public schools of books printed in small type. Small type is bad enough, but what can be worse than small German type!

